# Notes and News

**Published:** 1888-05-26

**Authors:** 


### Notes and News.

Collected and Communicated.

Their Royal Highnesses the Prince and Princess of Wales have consented to be present and to open the new buildings of the Great Northern Central Hospital on Monday, the 25th June. The hospital is situated in the Holloway Road, between the Nag's Head and Highgate Archway.

The Dowager Countess Castle Stuart laid the foundation of a convalescent home at Chelston, near Torquay, on the 16th inst.

The committee of the Seamen's Hospital have appointed Dr. John Anderson, C.I.E., to the post of visiting physician, rendered vacant by the resignation of Dr. Griffiths. Dr. Anderson, who has just retired from the medical staff of the army as Brigade Surgeon, lately served as surgeon to the Viceroy of India. He has had a vast experience in tropical disease, which ought to be of great service to him as physician to such a cosmopolitan institution as the "Dreadnought."

The annual general meeting of the Hospitals Association will be held in the large committee-room, Westminster Town Hall (close to St. James's Park Station), on Wednesday, May 30th, at five o'clock. Dr. T. Syer Bristowe, F.R.S. (the president), will preside, and deliver an address. It is expected that a large number of representatives of London and provincial hospitals will be present. Members of the Association are invited to bring any friends interested in the objects of the Association.

The St. Bartholomew's Medical Missionary Society, of which Dr. R. Thorne Thorne, F.R.C.P., is treasurer, is making efforts to add to its resources and to increase its usefulness. From every point of view medical missions are to be commended, and ought to be sustained. They bless even more than ordinarily both the students who belong to them, and the distant nations whom it is sought to teach and help. All good men and women, and particularly all old "Bart.'s" men and supporters, should feel specially bound to help.

A third list of old subscribers who have increased their annual subscriptions, and of new subscribers to the Richmond Hospital, has been published. The list is headed by the Hon. Algernon Grey Tollemache, who raises his subscription from forty guineas to fifty pounds. Many others have converted the hitherto unchangeable guinea into two guineas. A large number of hospitals might be delivered from the "Slough of Despond" by this simple process.

Mr. Charles Wesley, writing to the *St. James's Gazette*, complains of the disproportion between the sums paid to "the sisterhoods who farm out trained nurses," and the salaries received by the nurses themselves. He writes in the interest of the public, who pay what may be termed a "fancy price" for the services of a nurse, but we would support his contention in the interests of the nurses themselves, who certainly have a right to receive as payment the fair trade price of their services—a price decided, like all others, by honest competition in the open market—with only such deductions as are necessary to supply the funds for conducting the agency to which they belong. Without in the least intending it—actuated indeed by the noblest and most unselfish motives—some sisterhoods are "sweating" their nurses.

The business-like and energetic way in which the Hospital Saturday collections are made in Birmingham is proved by the fact that the sums received are counted and published every hour, and post cards, on which are printed the sums received at the same hours during the four last years are ready to be filled up with the amount that comes in on the latest occasion. Here is the comparative statement of five years, as despatched to us at ten p.m. on Saturday, the 12th :—

A good deal of talk has been indulged in for some time about the unnecessary quantity of alcohol consumed at the Devon and Exeter Hospital. At the quarterly meeting just held, the President, Mr. Trehawke Kekewich, brought the matter prominently before the governors. He said he had been at pains to inquire into the consumption of alcohol at the hospital, and it was only fair to say that it was not at all or very little greater than it had formerly been. No more alcohol was used than was thought necessary by the medical staff for the use of the patients. Mr. Ley confirmed this judgment, and stated that, as the result of his inquiries into the matter, he found that the average quantity of alcohol consumed in the London hospitals exceeded by one-third that which was found necessary at the Devon and Exeter Hospital. These explanations were regarded as highly satisfactory.

The annual meeting of the committee and subscribers of the Central London Throat and Ear Hospital, Gray's Inn Road, was held in the board-room of the institution last week, Captain Hutton (the chairman of the committee) presiding. The report showed that in the past year 5,845 new out-patients had been treated, involving 28,685 separate attendances, and that 340 patients had been admitted into the wards. These numbers were very largely in excess of those of any previous year, and the increase was such that it was often a matter of difficulty to accommodate within the building the press of out-patients daily in attendance ; and at all times a large number were waiting their turn for admission as in-patients. The income had been £1,877, a decrease of £13 compared with the receipts of the previous year ; and the expenditure had been £1,966. The actual liabilities amounted to £1,662, which sum included £1,000 due to the bankers, as the balance of an original mortgage of £3,500. This sum must be repaid before July next, and towards the extinction of this debt an earnest appeal is made.

THE following vacancies are announced this week, the amount of salary in each case being given after the name of the institution :—

**Resident Medical Officers.**

Brighton, Hove, and Preston Dispensary. House Surgeon.—£140 per annum.
Bristol Royal Infirmary. House Physician.—£100 per annum, with board, etc.
Chelsea Hospital for Women. Resident Medical Officer.—£60 per annum, with board.
Children's Hospital, Birmingham. Assistant Resident Medical Officer.—£40 per annum, with board.
Children's Hospital, Birmingham. Resident Medical Officer.—£80 per annum, with board.
Counties Asylum, Carlisle. Assistant Medical Superintendent.—£120 per annum, with board.
Fulham Union. Resident Medical Superintendent of Infirmary and Medical Officer of Workhouse.—£350 per annum.
Halifax Infirmary and Dispensary. Senior House Surgeon.—£80 per annum, with board.
Metropolitan Asylums Board Small pox Hospital Ships. Assistant Medical Officer.—Board, etc.
Metropolitan Hospital, Kingsland Road. Assistant House Surgeon.—£40 per annum, with board.
North London Consumption Hospital, Hampstead and London. Resident Medical Officer.—£50 per annum, with board.
North Staffordshire Infirmary. House Physician.—£100 per annum, with board, etc.
St. Peter's Hospital for Stone, etc., Henrietta Street, Covent Garden. House Surgeon.—£26 5s. per annum, with board, etc.
Victoria Hospital for Children, Chelsea. House Physician.—£50 per annum, with board.
Victoria Hospital for Children, Chelsea. House Surgeon.—£50 per annum, with board.

**Non-resident Medical Officers.**

Baltinglass Union. Medical Officer Dunlarin Dispensary.—£135 per annum, and fees.
City of London Hospital for Diseases of the Chest, Victoria Park, E.—Assistant Physician.
Gordon Hospital for Fistula, etc., Vauxhall Bridge Road. Assistant Surgeon.
King's College Hospital Assistant Surgeon.
London Temperance Hospital, Hampstead Road. Surgeon.
Lowestoft Friendly Societies Medical Institution. Surgeon.—£160 per annum, with fees.
Wellingborough and District Medical Institute. Medical Officer.—£280 per annum, with fees.
West London Hospital, Hammersmith Road, W. Assistant Surgeon.
Westport Union. Medical Officer, Westport No. 2 and Louisburgh No. 2 districts.—£39 per annum, and fees.

---

THE report of the Perth Sick Nursing Society gives a considerable amount of information about a kind of work which, now that the Queen has resolved to devote the Women's Jubilee Offering to the development of district nursing, is certain to attract much attention—the nursing of the sick poor at their own homes. The Perth Society has only two nurses on its staff; but these two paid 4,500 visits during the year, and thereby did much to alleviate the inevitable discomforts that sickness entails in the homes of the poor, where otherwise, it may be, only a helpless and ignorant child would have been available as nurse. It is satisfactory to see from the report that the Society are solicitous for the welfare of their nurses as well as for that of the poor, and have lately purchased a suitable home for them at a cost of £400.

---

AN extraordinary scene was witnessed at Wellingborough post-office the other morning, when, shortly after seven o'clock, a loud knocking was heard at the door. On this being opened a young woman rushed into the office and demanded to be allowed to send a telegram. On being told that she could not, she leaped over the counter, smashed the windows with her hands, broke the chandeliers, and, finally, tore every fragment of clothing off her body. No one was in the office at the time except the officials, who immediately fetched blankets and wrapped them round the woman. It was subsequently discovered that she was the daughter of a working man named Maddison. On Saturday night she slept away from home at a friend's house, and on Sunday morning got up, jumped out of window, and ran to the post-office. It is believed that she is suffering from religious mania.

THE Gorleston Cottage Hospital is a useful institution for the poor of a district whose inhabitants are chiefly dependent on the fishing industry, and are therefore peculiarly liable to accident and disease. Through the energy of the Hon. Secretary, Mr. Arthur W. Blake, of Thornville, Great Yarmouth, an out-patients' department has been added to the hospital, which will add greatly to its efficiency and usefulness.

---

A "HIGH-HANDED PROCEEDING," as it is called by a daily contemporary, is reported from the Victoria Park Chest Hospital. A young man who had died there is said to have been "dissected" in spite of the father's express prohibition. It may be assumed, since there is no medical school at the Victoria Park Hospital, that what really took place was a *post-mortem* examination. That we should suppose would hardly have been persevered with if it had been known at head-quarters that the father of the deceased decidedly objected. Those who rule in hospitals are seldom "high-handed" with the poor, and we should prefer to hear the statement of the hospital authorities before coming to any conclusion. We counsel our readers to exercise a similar patience.

---

THE Hamilton Association for Providing Trained Male Nurses was founded by Miss Hamilton in 1885. It is not a commercial institution, but was started (by means of voluntary subscriptions and donations) to benefit, firstly, the public, and secondly, the male nurses employed. By providing a corps of reliable men trained as nurses it is hoped that the public in general, and invalids in particular, will be benefited. We understand that, in addition to numerous private engagements, nurses have been supplied by the Association both to St. George's and Guy's Hospitals. The special advantages enjoyed by the men employed by the Association are numerous, one of the most important being the very small deduction from their wages which is made, the largest amount deducted being 10 per cent., and in some cases only 5 per cent. is taken off. Nurses thus practically retain their own earnings. As a nursing institution for the supply *solely* of *male* nurses we believe the Hamilton Association is unique, and we can speak most cordially of the skill, kindliness, and efficiency of the nurses supplied by it, who, as a rule, deserve the praise conveyed in Mr. Gilbert's quaint, but true, paradox, "Staunch as a woman, tender as a man." The head-quarters of the Association are at 69, South Audley Street, W.

---

MILTON affirms that "They also serve who only stand and wait." It is equally true that they also give to hospitals who only count the pence given by working men. The counters at Liverpool have had their work unnecessarily increased by the carelessness of many of those who have been in charge of the workshop and factory boxes. There are about a thousand boxes in the different localities, and if all these had been returned at once after notice given, those who volunteered to count the pence would have been able to get through their arduous task within a reasonable time. But, notwithstanding an urgent notice, it was found that not more than two hundred of the thousand boxes were sent in at once. Of course, those who are in charge cannot be accused of anything worse than carelessness, but carelessness is hardly excusable under such circumstances. We call special attention to what has taken place at Liverpool, because we think it not unlikely that the same kind of thing happens in many other towns. Will not working men who have charge of the boxes at their respective shops and factories make a point of facilitating the work of counting by sending in the contributions as soon as asked? They cannot have any idea of the time and trouble involved in the counting of vast heaps of pence and halfpence.

